# Digital Dentistry: Transformation of Oral Health and Dental Education with Technology

**DOI:** 10.1055/s-0043-1772674

**Published:** 2023-09-20

**Authors:** Zohaib Khurshid

**Affiliations:** 1 Department of Prosthodontics and Dental Implantology, College of Dentistry, King Faisal University, Al-Ahsa, Kingdom of Saudi Arabia; 2 Department of Anatomy, Center of Excellence for Regenerative Dentistry, Faculty of Dentistry, Chulalongkorn University, Bangkok, Thailand

## Aspects of Digital Dentistry

Without a doubt, dentistry is quickly modernizing and transitioning to digital technology. In the past, taking dental impression, making dental models, and assembling dental laboratory prostheses took a lot of time. However, the advent of the intraoral scanner, digital radiography, computer-aided design (CAD)/computer-aided manufacturing (CAM), 3D printers, and artificial intelligence (AI) software revolutionized the field of dentistry as we know it today. 1 2 3 Precision and accuracy are two of digital dentistry's key benefits. Cone beam computed tomography (CBCT) and digital radiography are two examples of digital imaging technologies that enable dentists to get extremely precise and detailed pictures of the teeth and surrounding tissues. 3 The utilization of these data allows for very precise and predictable treatment planning and execution. The effectiveness and quickness with which dental restorations may be made is another advantage of digital dentistry. With the use of CAD/CAM technology, dental restorations may be designed and created in a single session, doing away with the need for further visits and interim restorations. 4 Increased precision and accuracy, quicker production times, and more personalization options are all benefits of digital dentures. They also eliminate the requirement for traditional impression materials, which some patients could find painful. 5 Due to their reduced waste generation and resource usage, digital dentures may also be more ecologically friendly than conventional ones.

## Patient Education and Communication

Additionally, improved patient education and communication are provided by digital dentistry. Dental professionals may more easily and effectively communicate treatment alternatives to patients by using digital imaging and planning tools to show them their dental condition. Over traditional dentistry, digital dentistry has a number of benefits. Higher precision and accuracy, shorter treatment durations, more patient comfort, more effective teamwork and communication, and greater patient education are a few of these benefits. By lowering waste, energy use, chemical use, transportation emissions, and paper use, digital dentistry has the potential to lessen the environmental impact of dental care. 6 Dental clinics may take strides toward improved environmental sustainability by implementing digital dentistry technology. By enhancing patient outcomes, boosting efficiency, improving patient communication and happiness, encouraging teamwork among dental professionals, and improving record keeping, digital dentistry may help both private practice and dental hospitals.

## Limitation of Digital Dentistry

While digital dentistry offers numerous benefits, it is important to acknowledge potential limitations and challenges associated with its implementation. One limitation is the initial cost of adopting digital technologies, which can be a barrier for some dental practices. Additionally, the learning curve associated with mastering new digital tools and techniques may require additional training and time for dentists and dental staff. Moreover, concerns regarding data security and privacy need to be addressed to ensure the protection of patient information in digital systems. Another limitation is the reliance on technology, as technical malfunctions or system failures could disrupt dental workflows and potentially compromise patient care. Furthermore, not all dental procedures can be fully replaced by digital technologies, and there may still be a need for traditional approaches in certain cases. By acknowledging these limitations and addressing potential concerns, a more balanced and comprehensive perspective on digital dentistry can be presented.

## Digital Dentistry in Dental Education

Digital dentistry has revolutionized dental education and transformed oral health practices. The integration of digital resources, such as simulation software and virtual reality technologies, within dentistry schools has significantly enhanced the effectiveness and efficiency of instruction for students. 7 Moreover, the utilization of digital imaging and treatment planning technologies has greatly improved patient knowledge and communication, leading to superior oral health outcomes. Innovative techniques like CAD/CAM and laser dentistry, which are central to digital dentistry, offer precise, effective, and comfortable treatments, ultimately enhancing patient satisfaction and overall outcomes. Furthermore, the adoption of digital dentistry has the potential to reduce waste, energy consumption, and chemical usage, thus contributing to environmental preservation. Undoubtedly, the profound impact of digital dentistry has the capability to revolutionize dental education and elevate the oral health results for patients.

## Conclusion

In conclusion, the advent of digital dentistry has ushered in a new era of advancements in both oral health and dental education. Through its utilization of precise imaging, efficient treatment planning, and personalized restorations, digital dentistry has successfully transformed traditional practices. Moreover, the integration of digital technologies has led to substantial improvements in patient education, fostering enhanced communication and understanding between dental professionals and patients. Additionally, digital dentistry offers significant environmental benefits by reducing waste, energy consumption, and chemical usage, aligning with sustainable practices to preserve our planet.

As we look ahead, digital dentistry stands as a powerful force that shapes the future of dental care. Its continuous evolution and innovative techniques pave the way for improved diagnostic accuracy, streamlined workflows, and enhanced treatment outcomes. By embracing digital dentistry, dental professionals can provide patients with more precise, effective, and personalized treatments, ultimately leading to greater patient satisfaction and improved oral health results.

In summary, digital dentistry represents a paradigm shift in oral health care, revolutionizing the field and propelling it into a future of cutting-edge advancements. Through its transformative potential, it empowers dental professionals to deliver exceptional care, elevating patient experiences, and paving the way for a brighter future in dental education and oral health.

## References

[1] Wimmer T Gallus K Eichberger M Stawarczyk B Complete denture fabrication supported by CAD/CAM J Prosthet Dent 2016 115 05 541 546 26774323 10.1016/j.prosdent.2015.10.016

[2] Wagner S A Kreyer R Digitally fabricated removable complete denture clinical workflows using additive manufacturing techniques J Prosthodont 2021 30(S2):133 138 33988280 10.1111/jopr.13318

[3] Fang J H An X Jeong S M Choi B H Digital intraoral scanning technique for edentulous jaws J Prosthet Dent 2018 119 05 733 735 28888413 10.1016/j.prosdent.2017.05.008

[4] Anadioti E Kane B Soulas E Current and emerging applications of 3D printing in restorative dentistry Curr Oral Health Rep 2018 5 02 133 139

[5] Fang J H An X Jeong S M Choi B H Development of complete dentures based on digital intraoral impressions: case report J Prosthodont Res 2018 62 01 116 120 28625663 10.1016/j.jpor.2017.05.005

[6] Duane B Harford S Steinbach I Environmentally sustainable dentistry: energy use within the dental practice Br Dent J 2019 226 05 367 373 30850795 10.1038/s41415-019-0044-x

[7] Imran E Adanir N Khurshid Z Significance of haptic and virtual reality simulation (VRS) in the dental education: a review of literature Appl Sci (Basel) 2021 11 21 10196

